# Assessment of neuropathic pain in leprosy patients with relapse or treatment failure by infrared thermography: A cross-sectional study

**DOI:** 10.1371/journal.pntd.0009794

**Published:** 2021-09-23

**Authors:** Liliane Marques de Pinho Tiago, Diogo Fernandes dos Santos, Douglas Eulálio Antunes, Letícia Marques Pinho Tiago, Isabela Maria Bernardes Goulart

**Affiliations:** 1 National Reference Center for Sanitary Dermatology and Leprosy, Clinical Hospital, Federal University of Uberlandia, Uberlândia, Brazil; 2 Graduate Program in Health Sciences, Faculty of Medicine, Federal University of Uberlandia, Uberlândia, Brazil; 3 Graduate Program in biomedical engineering, Faculty of Electrical Engineering, Federal University of Uberlandia, Uberlândia, Brazil; Emory University, UNITED STATES

## Abstract

**Background:**

Neuropathic pain (NP) is one of the main complications of leprosy, and its management is challenging. Infrared thermography (IRT) has been shown to be effective in the evaluation of peripheral autonomic function resulting from microcirculation flow changes in painful syndromes. This study used IRT to map the skin temperature on the hands and feet of leprosy patients with NP.

**Methodology/Principal findings:**

This cross-sectional study included 20 controls and 55 leprosy patients, distributed into 29 with NP (PWP) and 26 without NP (PNP). Thermal images of the hands and feet were captured with infrared camera and clinical evaluations were performed. Electroneuromyography (ENMG) was used as a complementary neurological exam. Instruments used for the NP diagnosis were visual analog pain scale (VAS), Douleur Neuropathic en 4 questions (DN4), and simplified neurological assessment protocol. The prevalence of NP was 52.7%. Pain intensity showed that 93.1% of patients with NP had moderate/severe pain. The most frequent DN4 items in individuals with NP were numbness (86.2%), tingling (86.2%) and electric shocks (82.7%). Reactional episodes type 1 were statistically significant in the PWP group. Approximately 81.3% of patients showed a predominance of multiple mononeuropathy in ENMG, 79.6% had sensory loss, and 81.4% showed some degree of disability. The average temperature in the patients’ hands and feet was slightly lower than in the controls, but without a significant difference. Compared to controls, all patients showed significant temperature asymmetry in almost all points assessed on the hands, except for two palmar points and one dorsal point. In the feet, there was significant asymmetry in all points, indicating a greater involvement of the lower limbs.

**Conclusion:**

IRT confirmed the asymmetric pattern of leprosy neuropathy, indicating a change in the function of the autonomic nervous system, and proving to be a useful method in the approach of pain.

## Author summary

Pain has been shown to be a significant problem for leprosy patients and may be of nociceptive origin due to tissue inflammation, which occurs during reactional episodes mediated by the immune system, or neuropathic due to leprosy affecting the somatosensory system. It is important to differentiate neuropathic pain from chronic neuritis pain because the clinical implications and treatment are different. Multidrug therapy does not seem to prevent the occurrence of neuropathic pain, which is associated with low indices of quality of life and the general state of health. Infrared thermography is a complementary imaging test that is still growing and can be used in monitoring and determining the prognosis of patients in the health area. The thermographic examination records the abnormal thermal distribution and temperature differences in circulation alterations, which are not noticeable in the subjective evaluation of pain. We believe that the use of thermography as a complementary exam in the study of pain in leprosy neuropathy can assist in the definition of conduct protocols, which will enable the tracking and referral of patients to specific sectors in specialized health units, thereby changing the direction of the disease.

## Introduction

Leprosy is a chronic infectious disease caused by the bacillus *Mycobacterium leprae* [1], which, due to its high affinity for peripheral nerves, has been reported as one of the most common causes of treatable peripheral neuropathy in the world [2]. Peripheral nervous system involvement occurs by two main factors, including the bacillus’s predilection for Schwann’s cell and reactions mediated by the host immune system [3]. This neural impairment often leads to changes in sensory, motor, and autonomic function [4]. Leprosy peripheral neuropathy may occur before, during, or after treatment with multidrug therapy (MDT) [5].

Pain has been shown to be a significant problem in leprosy neuropathy, and may be of nociceptive origin due to neuritis, which occurs during reactional episodes, neuropathic due to the involvement of the somatosensory system, or mixed (nociceptive and neuropathic) [6,7]. According to studies, neuropathic pain (NP) has been shown to be one of the most common late complications of leprosy [8,9], which may clinically manifest continuously or intermittently and occurs in a single or in several locations [10]. The prevalence of NP in leprosy has been described as 45% in China, 21% in India, 11% in Ethiopia, and 56% in Brazil; this variation in prevalence is due to the use of different study models, clinical forms of the selected patients, and screening tools [6,10–12]. As pain is a subjective symptom, difficult to measure, and involves physical and psychic aspects, making the diagnosis is sometimes a challenge [7]. It is important that NP is well diagnosed and adequately treated, since it is associated with low indices of quality of life and general health status [8,5].

In the absence of biomarkers or other gold standard examination, the diagnosis is initially clinical [13]. Validated clinical instruments for pain screening, such as Neuropathic Symptoms and Signs (LANSS), Douleur neuropathique en 4 questions (DN4), and painDETECT are often used [14].

There are some quantitative assessment methods available that can assist in the approach of NP, such as infrared thermography (IRT), which allows the mapping of the skin surface temperature, capturing changes in the microcirculation blood flow [15]. According to the previous study, there is a good correlation between skin temperature and cutaneous nervous activity, showing that skin temperature is a good predictor of sympathetic activity [16]. The sympathetic nervous system is the main regulator of cutaneous thermal emission [17]. Therefore, IRT allows to evaluate the abnormal thermal distribution and temperature differences caused by changes in the cutaneous peripheral circulation in various pathologies [18]. IRT has been used for the early and differential diagnosis of several pathological syndromes, as well as in painful syndromes, such as Complex Regional Pain Syndrome, Myofascial, Post Traumatic, Fibromyalgia, and neuropathic pain, and in inflammatory diseases of the skeletal muscle system [18,19].

The present study used IRT to assess the function of the peripheral autonomic nervous system by mapping the skin temperature of the hands and feet of leprosy patients who were undergoing treatment for relapses or treatment failure. Considering the magnitude of neuropathic pain in leprosy, this study aims to describe thermographic findings in leprosy patients, evaluating the association of these findings with the presence of neuropathic pain, as autonomic impairment may accompany this clinical condition.

## Methods

### Ethics statement

The Committee of Ethics in Research from the Federal University of Uberlandia (UFU)/MG approved this study (CAAE: 60427816.4.0000.5152). The researchers explained the aim of the study to the participants, and those who agreed to participate in the study signed the free and informed consent form.

### Study design and subjects

**Fig 1:**
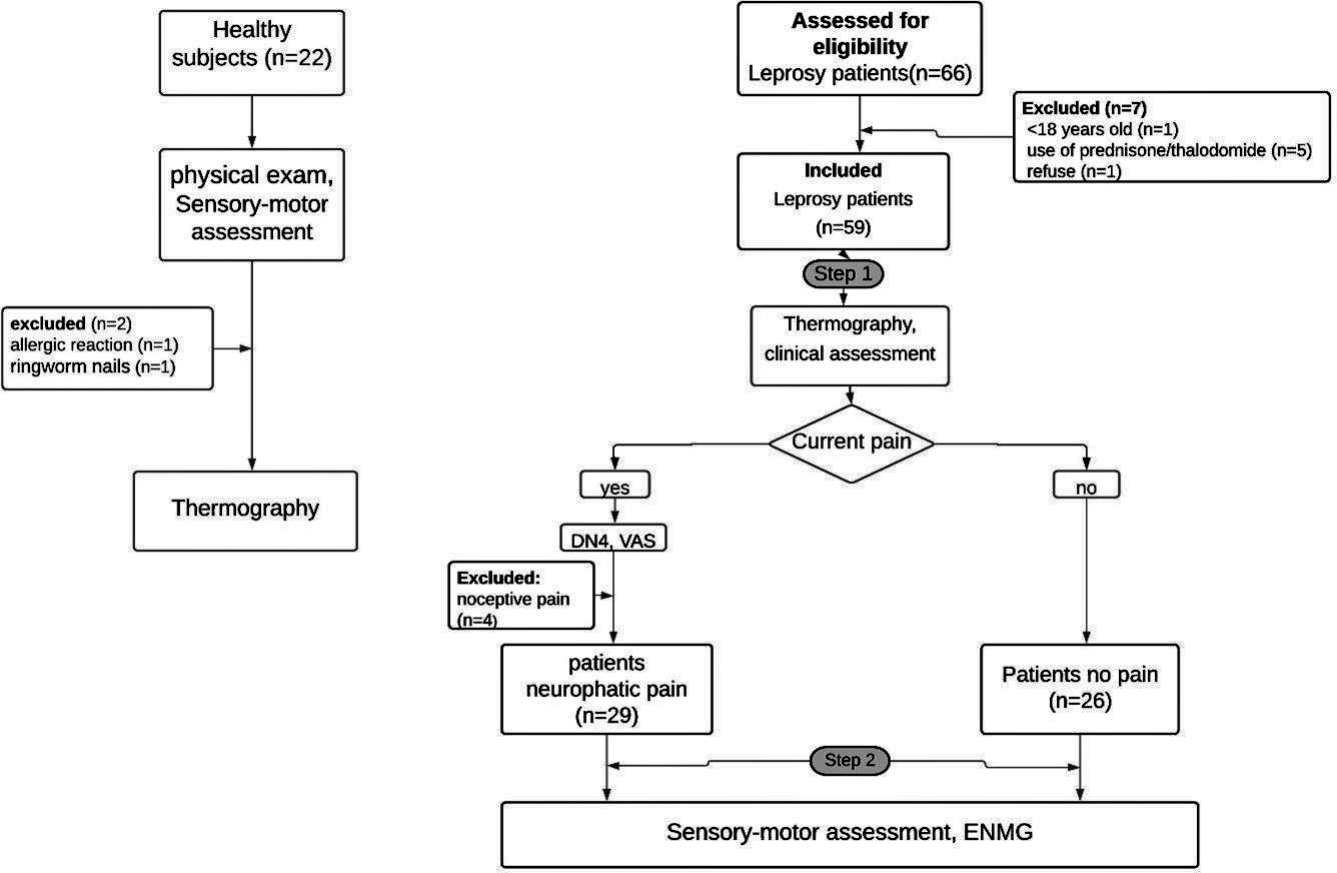
Flowchart of data collection and subject selection.

This cross-sectional study was conducted from January 2018 to December 2019 at the National Reference Center for Sanitary Dermatology and Leprosy (CREDESH), Clinical Hospital, Federal University of Uberlandia, located in Uberlandia/MG, Brazil. Patients (>18 years old) diagnosed with leprosy relapse or failure of previous treatment were eligible for this study. Clinical criteria were used for the diagnosis of leprosy relapse or treatment failure as follows: 1) Presence of active skin lesions; 2) New areas with change in sensitivity; 3) Neurological abnormalities, persistent neuritis, or reactional outbreak unresponsive to clinical treatment. Laboratory criteria used included: 1) Presence and characteristics of bacillus according to the bacilloscopic index in dermal scraping and skin biopsy; 2) Presence and quantification of bacillary DNA by qPCR (threshold cycle result) in dermal scraping and skin biopsy; 3) Maintenance or increase of the ELISA IgM anti-PGL-I index. Regarding the time to relapse, it was considered as greater than or equal to five years from the time of discharge from the last treatment. Regarding treatment failure, patients who had no clinical and laboratory improvement were defined as stated above, at the time of discharge from MDT for 24 months or alternative ROM regimen (rifampicin 600 mg + ofloxacin 400 mg + minocycline 100 mg) in monthly supervised doses for 24 months [20]. This study excluded patients who had reactional states, plantar ulcers, vasculopathies, and/or peripheral neuropathies of other etiologies (diabetes mellitus, HIV, alcoholism, etc.), including toxic and dapsone-related neuropathy. A total of 55 leprosy patients were selected and distributed into two groups: Patients with NP (PWP) and patients without pain (PNP). Patients with nociceptive pain were excluded from this study. The selection of the sample and the study design are illustrated in Fig 1.

To compare epidemiologic data and thermal measurements, 22 healthy individuals who had no contact with leprosy patients were invited to participate in this study as a control group. Their health status was confirmed by physical exam, and those with a normal sensory and motor assessment were included, while individuals with suspected systemic or localized diseases were excluded. The final sample consisted of 20 healthy individuals (Fig 1).

### Thermal assessment

The thermal images of the hands and feet of healthy individuals and leprosy patients were captured using a thermal camera. To perform the IRT, the patients were instructed to prepare according to the protocol of the American Academy of Thermology [21], which consists of not performing physiotherapy, acupuncture, physical activity, or an electrodiagnostic test in the 24-hour period prior to the exam; not consuming caffeine products or nicotine in the 4-hour period prior to imaging, and not using cosmetics (skin lotions, sun screens, deodorants, etc.) on the hands or feet on the day of the exam.

On the day of the examination, the individuals were placed in an air-conditioned room without air currents or a heat source. The lighting of the room was made with fluorescent lamps to prevent the heating of the room. The temperature and humidity were monitored using the digital thermo-hygrometer Incoterm model: 7666.02.0.00. The room temperature was kept at 23°C ± 1°C and relative humidity at 55.5% ± 3.5%, and the participants remained seated and barefoot, resting in this environment for 15 minutes for acclimatization. A qualified physician captured the images using an infrared camera, with high spatial resolution of 320 x 240 pixels, calibrated with thermal sensitivity from 0.045°C to 30°C, frequency of 60 Hz, and having the ability to adjust the emissivity to 0.98. The camera was positioned on a tripod at a distance of 67–70 cm from the participant, and IT was captured from the hands (palm and dorsum) and feet (dorsum and sole) bilaterally. The IT obtained were saved in Joint Photographic Experts Group (JPEG) format and analyzed using thermography software.

**Fig 2:**
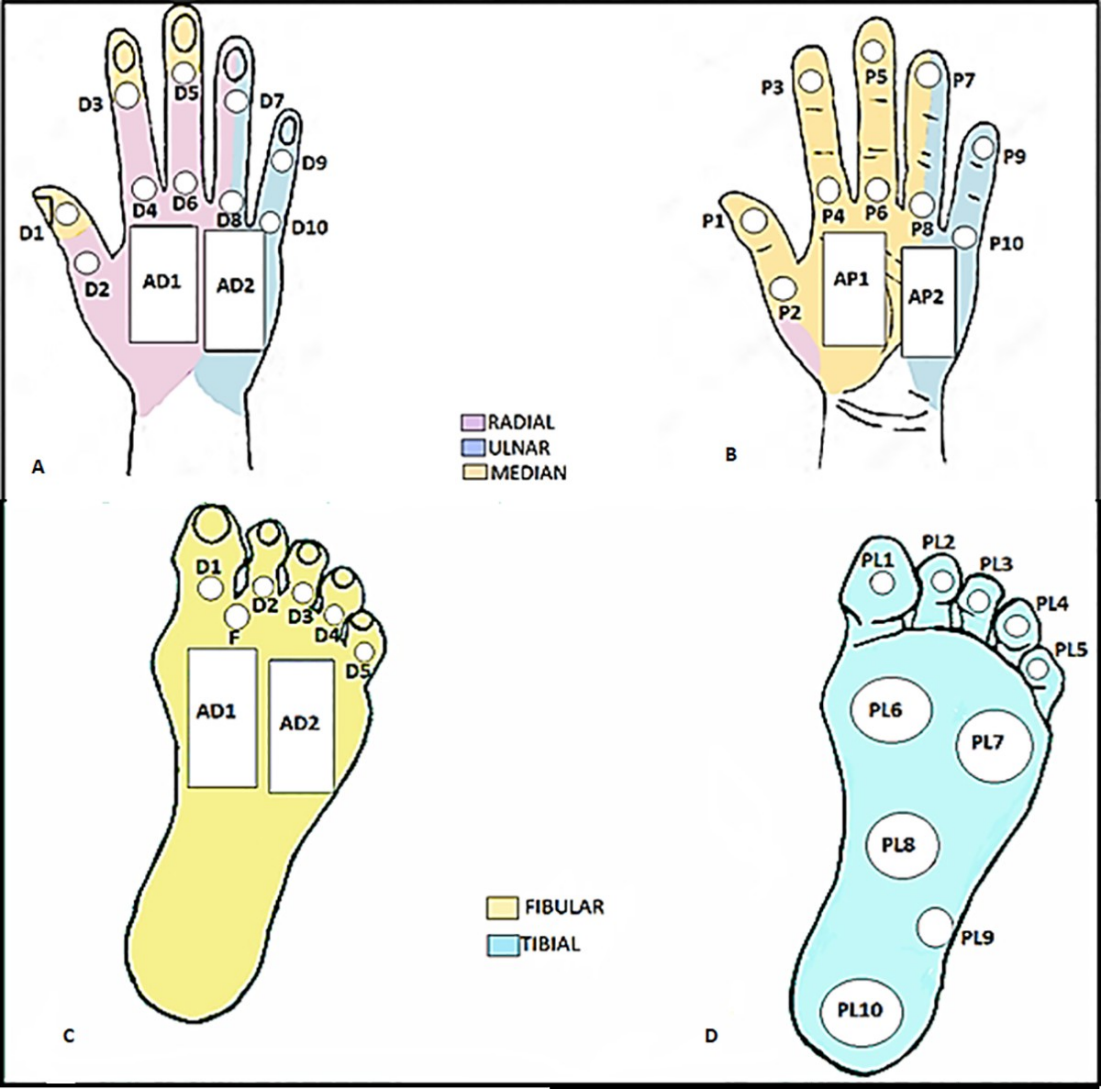
Region of interest of hands and feet. (A) Dorsum of the hand; (B) Palm of the hand; (C) Dorsum of foot; (D) Plantar of foot.

Regions of interest (ROIs) were defined for quantitative temperature evaluation, 24 in the hands, back, and palm, and 17 in the feet, plant, and back (Fig 2). The ROIs coincided with the areas of innervation of the peripheral nerves frequently affected in leprosy, which are the ulnar, median, radial, common fibular, and tibial nerves. To assess the temperature in the peripheral innervation territories of the skin, ROIs were analyzed separately and grouped by neural area. Regarding the measurements in the neural area (grouped ROIs), the mean of the points referring to the dermatome of each peripheral nerve was calculated.

The ROIs defined in each neural area were: 1) Median nerve: P1, P2, P3, P4, P5, P6, AP1, D1, D3, and D5; 2) Ulnar nerve: P9, P10, AP2, D9, D10, and AD2; 3) Radial nerve: D2, D4, D6, and AD1; 4) Tibial nerve: PL1, PL2, PL3, PL4, PL5, PL6, PL7, PPL8, PL9, and PL10; 5) Fibular nerve: D1, D2, D3, D4, D5, F, AD1, and AD2 (Fig 2). Points P7, P8, D7, and D8 were not included in the neural areas due to the mixed innervation.

For statistical comparison, the average temperatures of the ROIs (isolated and grouped) and the temperature difference (ΔT = Tright—Tleft) between the contralateral hands and feet were calculated. The ΔT was used to assess asymmetry and detect possible dysfunctions.

### Clinical evaluation

The patients underwent clinical evaluation during which sociodemographic (age, gender) and clinical data were collected: World Health Organization (WHO) and the Ridley-Jopling classification [22], previous MDT treatment, presence of pain, time of onset of pain, and previous leprosy reactions. In the control group, only sociodemographic data were collected.

Participants in the PWP group were asked to locate the anatomical region of their pain, and the degree of pain intensity was assessed using the visual analog pain scale (VAS), where 0 = no pain and 10 = maximum pain [23]. The intensity of pain was interpreted and classified as: 0: no pain, 1–3: mild pain, 4–7: moderate, 8–10: severe. The presence of NP was evaluated using the *Douleur Neuropathique en 4 Questions* (DN4) [24], a universal instrument that has been validated in Portuguese [25], composed of seven items related to symptoms and three related to the clinical examination, totaling ten points. Each positive response was given a point. When the total score was greater than or equal to 4, the pain was classified as NP, and below this value, it was classified as nociceptive.

### Sensory-motor assessment

The leprosy patients were submitted to the assessment of the peripheral sensory-motor function by the physiotherapy team at the CREDESH. In the motor assessment, functional tests were performed in the muscle groups of abductor pollicis brevis and lumbricals (first and second) for the median nerve; first dorsal interosseous, abductor digiti minimi, and lumbricals (third and fourth).for the ulnar nerve; common extensor of the fingers and radial carpal for the radial nerve; and the muscles tibial anterior, extensor digitorum longus, and extensor hallucis longus innervated by the deep fibular nerve. The Medical Research Council Scale was used with a graduation of 0–5, considered as grade 0 = paralysis and 5 = normal strength, and any change in function < 4 in one or more muscle groups was considered abnormal [26].

The sensory evaluation was performed using the six Semmes-Weinstein filaments, which exert forces of 0.05 g, 0.2 g, 2g, 4g, 10g, and 300 g when applied to the skin, and the tested points coincided with the ROIs evaluated. Sensory loss was considered as the absence of positive response to filaments of 0.2 g in the hands and 2g in the feet [27].

### Electroneuromyography

Electroneuromyography was performed on the participants in the PWP and PNP groups to assess neuropathy, by the neurophysiologist of CREDESH. The device used in the examination was the MEB 4200K (NIHON-KODHEN) and the techniques and configurations of the examination were standardized in the previous study. The sensory nerves evaluated were the median, ulnar, dorsal cutaneous of the hand, radial, sural, superficial fibular, and medial plantar bilaterally, and the motor nerves were the median, ulnar, deep fibular, and tibial bilaterally. The electroneuromyography was classified according to the neurophysiological pattern in mononeuropathy: presence of only one altered nerve or asymmetric multiple mononeuropathy and presence of two or more altered nerves [28].

### Sample size calculation

It was calculated the sample size of this study using the software G*Power (version 3.1.9.2, for windows). One-way ANOVA fixed effects were performed by means of priori analysis with the effect size of 0.37, obtained from a pilot study concerning thermography (not published), alpha err probability of 5% (0.05), power of test (1-β err probability) 0.80 (80%) and the number of 3 groups. Thus, the total sample size, considering the above parameters, was 75 individuals.

### Statistical analysis

The *D’Agustino-Pearson test was used* to evaluate the normality of the values referring to the temperatures of each cutaneous region and the values of ΔT. The nonparametric distribution variables were normalized by the Log 10 transformation when necessary. The Binomial test evaluated the association between clinical/sociodemographic variables and patient groups with or without pain.

*The student’s t-test*, for paired samples, was used in the comparison between the average temperatures of the upper limbs right and left. *Mann-Whitney test* was used in the comparisons between the sum of the average of the stations for each ΔT referring to the differences in temperatures by cutaneous region obtained for different groups.

The statistical program used was the Statistical Package for Social Sciences version 22 (IBM, Armonk, NY, USA), with a significance level of 5% for all analyses.

## Results

Fifty-five leprosy patients were evaluated, with 29 patients in the PWP group and 26 patients in the PNP group. There was no statistical difference between the PWP group (46.75 ± 11.4 years; 58.6% (17/29) male), PNP group (48 ± 13.44 years: 61.5% (16/26) male), and healthy subjects (43.05 ± 14.74 years; 40% (8/20) male) in relation to age and gender (p>0.05). The control group presented normal sensory-motor function, being the inclusion criteria for this group. The clinical data of the PWP and PNP groups are shown in Table 1. There was also no significant difference between the leprosy patient groups in relation to the number of relapses or treatment failure of the disease, operational classification, date of onset, and type of current treatment.

**Table 1:** Clinical characteristics of the patients with and without neuropathic pain.

Variables	PWP	PNP	
	n = 29	n = 26	p-value
**Relapse**	23 (79.3%)	20 (76.9%)	
**Treatment Failure**	6 (20.7%)	6 (23.1%)	0.8305
**Operational**			
**classification**			
MB	23 (79.3%)	22 (84.6%)	
PB	6 (20.7%)	4 (15.4%)	0.6106
**Clinical Form**			
Tuberculoid	1 (3.5%)	0	
Borderline-tuberculoid	9 (31%)	10 (38.5%)	0.5631
Borderline-borderline	6 (20.7%)	1 (3.8%)	0.0613
Borderline-lepromatous	4 (13.8%)	0	
Lepromatous	9 (31%)	15 (57.7%)*	0.0466
**Current treatment**			
ROM	29 (100%)	25 (96.2%)	
Other	0	1 (3.8%)	0.2865
**Relapse time**			
*at* 6 months	20 (69%)	19 (73.1%)	
< 6 months	9 (31%)	7 (26.9%)	0.7375
**Leprosy reactions**			
Type 1	18 (57.6%)*	8 (30.8%)	0.0203
Type 2	11 (42.4%)	14 (53.8%)	0.2366
No	0	4 (15.4%)	
**Electroneuromyography**			
normal	0	6 (23.1%)	
mononeuropathy	2 (6.9%)	1 (3.8%)	0.6189
multiple mononeuropathy	27(93.1%)*	19 (73.1%)	0.0450
**sensory function**			
normal	3 (10.4%)	8 (30.8%)	
abnormal	26 (89.6%)	18 (69.2%)	0.0587
**motor function**			
normal	9 (31%)	19 (73.1%)	
abnormal	20 (69%)*	7 (26.9%)	0.0018
**Who disability grade**			
0	6 (20.7%)	11 (42.3%)	
1	12 (41.4%)	11 (42.3%)	0.0833
2	11 (37.9%)	4 (15.4%)	0.9444

PWP: patients with pain, PNP: patients no pain, MB: multibacillary, PB: paucibacillary.

n is a total number of cases; % is a percentage of cases.

WHO: World Health Organization, ROM: rifampin, ofloxacin, minocycline.

*p was considered significant when p< 0.05.

The previous reactional episodes type 1 were statistically significant in the PWP group (p = 0.020). The ENMG showed a predominance of multiple mononeuropathy in the patient groups, with a significant difference in the PWP and PNP groups (p < 0.045). Most individuals presented sensory loss and some degree of disability, but there was no significant difference between the PWP and PNP groups. There was a greater loss of motor function in the PWP group (p = 0.001) (Table 1).

In the PWP group, 93.1% (27/29) of patients had NP and 6.9% (2/29) mixed pain. The prevalence of NP was 52.7% (29/55), considering individuals who had mixed pain. The most frequent DN4 items in individuals with NP were numbness and tingling 86.2% each (25/29), electric shocks 82.7% (24/29), hypoesthesia to touch 75.8% (22/29), pins and needles 72.4% (21/29), and burning 68.9% (20/29), and the least frequent were brushing/painful cold with 37.9% each (11/29), hypoesthesia to prick 34.5% (10/29), and Itching 27.6% (8/29). All patients with NP had chronic pain, and as for the periodicity of pain, 62.1% (18/29) of the individuals reported that the pain was intermittent and 37.9% (11/29) reported that the pain was continuous.

**Fig 3:**
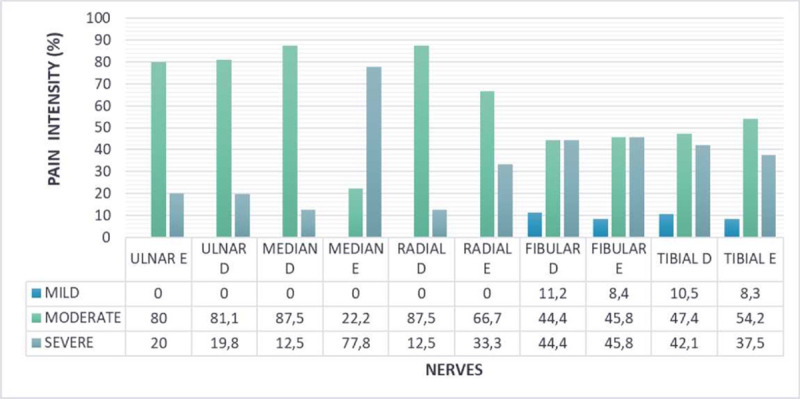
Percentage of pain intensity (VAS) in nerves of patients with neuropathic pain.

The patients reported NP in the innervation territory of the following nerves: 89.6% (26/29) left tibial, 75.9% (22/29) left fibular, 58.6% (17/29) right tibial, 62.1% (18/29) right fibular, 55.2% (16/29) left ulnar, 41.4% (12/29) right ulnar, 34.5% (10/29) left radial and median, and 31.03% (9/29) right radial and median. Only 13.8% (4/29) of the patients with NP reported pain in only one nerve, and 86.2% (25/29) had two or more nerve involvement. Pain intensity assessed by VAS showed that 93.1% (27/29) of patients with NP had moderate/severe pain. The intensity of peripheral nerve topography is represented in Fig 3.

**Fig 4:**
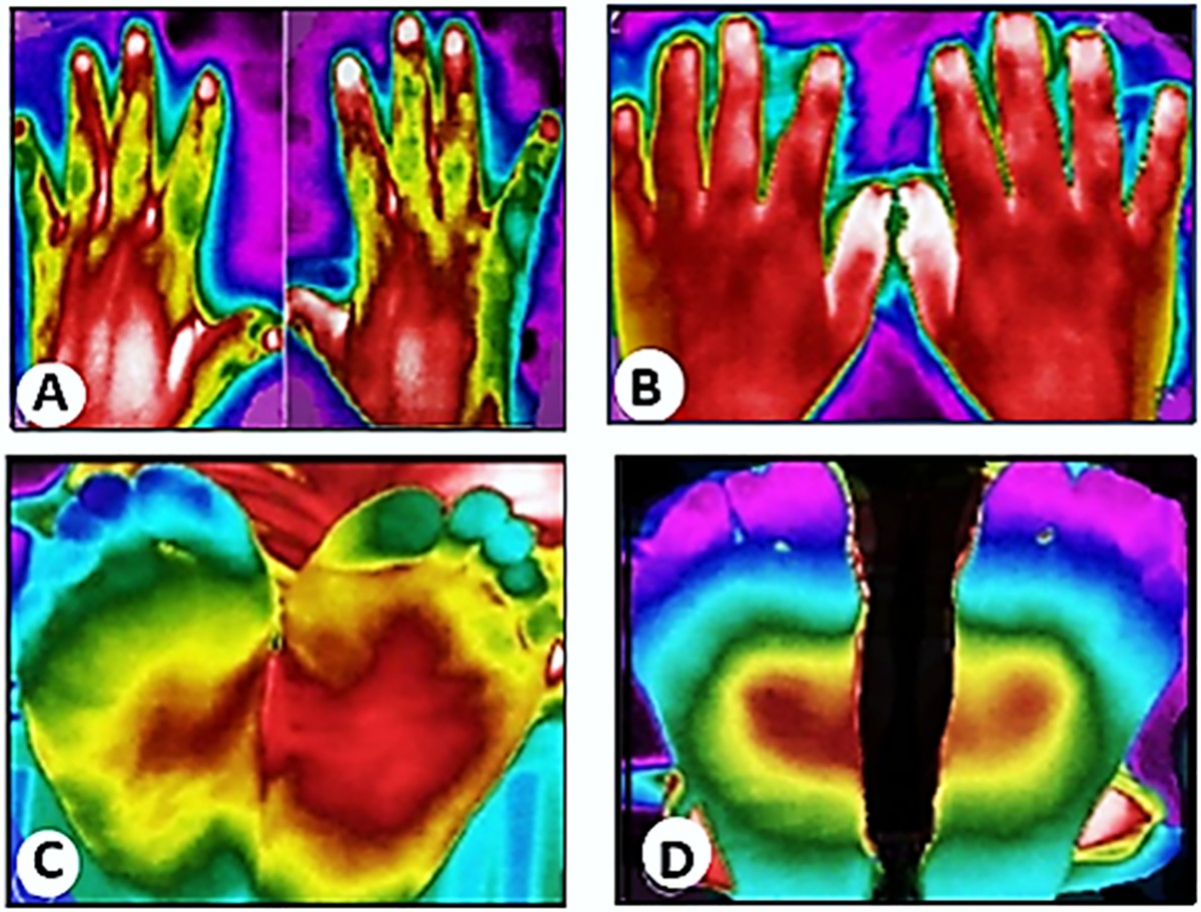
Infrared thermal imaging. (A) Thermal image of the dorsal region of the hands of patient with leprosy; (B) Thermal image of dorsal region of the hands of a healthy individual; (C) Thermal image of feet of a patient with leprosy; (D) Thermal image of the plantar region of feet of a healthy individual.

Comparisons of the mean temperature of each individual with that of the contralateral side was performed, and the ΔT measurements were compared between the group of patients (PWP and PNP) and healthy controls. The average temperatures of the ROIs and the neural areas ranged from 31.69°C to 33.74°C in the hands and 29.5°C to 32.06°C in the feet, in the group of patients (PWP and PNP). There was a significant mean temperature difference in P4, P5 and P10 in the hands and PL7, PL8, D2, and D3 in the feet of the group of leprosy patients. In the healthy controls, the average temperature was between 32.34°C to 33.71°C in the hands and 30.38°C to 32.15°C in the feet, but only ROI P5 had a significant difference in this group (S1 Table). In all groups, the average temperature in the lower limbs was lower than that of the upper limbs (Table 2). To assess symmetry, the ΔT was also calculated and compared among the groups of patients and healthy controls. There was a significant difference between the ΔT of almost all the evaluated points (p < 0.05) in hands, including in the neural areas, except for the palm points (AP1 and AP2) and one point on the back of the hands (AD1) (Table 2). In the feet, there was a significant difference in all the ROIs and neural areas analyzed, indicating a greater involvement in the lower limbs. In the hands of all patients, the median ΔT ranged from 0.2°C to 0.7°C, and in the feet, the median ranged from 0.4°C to 0.7°C. In the hands of the control group, the median ΔT ranged from 0.15°C to 0.3°C, and in the feet, it ranged from 0.15°C to 0.25°C. IRT detected the asymmetry of the patients’ hands and feet when compared with subjects in the control group (Fig 4).

**Table 2:** Comparison of ΔT in ROIs and neural areas of the hands between the group of patients and healthy controls.

	LEPROSY PATIENTS (n = 55)	HEALTHY CONTROLS (n = 20)	
ROI	ΔT	ΔT	
	median (Q1-Q3)	median (Q1-Q3)	*p value*
**HANDS**			
P1	0.6 (0.3–0.9)	0.2 (0.1–0.2)	<0.001
P2	0.5 (0.2–0.7)	0.2 (0.1–0.3)	0.006
P3	0.4 (0.2–0.9)	0.2 (0.1–0.4)	<0.001
P4	0.4 (0.2–0.7)	0.2 (0.1–0.3)	0.008
P5	0.5 (0.3–0.9)	0.15 (0.1–0.2)	<0.001
P6	0.4 (0.2–0.7)	0.2 (0.1–0.3)	0.007
P7	0.5 (0.2–0.9)	0.25 (0.1–0.4)	0.002
P8	0.5 (0.2–0.9)	0.2 (0.2–0.3)	0.002
P9	0.6 (0.2–1.1)	0.2 (0.12–0.3)	<0.001
P10	0.3 (0.2–1.0)	0.2 (0.1–0.3)	0.004
AP1	0.2 (0.1–0.5)	0.2 (0.12–0.3)	0.525
AP2	0.3 (0.1–0.7)	0.25 (0.2–0.3)	0.154
D1	0.4 (0.2–1.0)	0.3 (0.2–0.37)	0.025
D2	0.4 (0.1–0.9)	0.2 (0.2–0.3)	0.013
D3	0.4 (0.1–1.0)	0.2 (0.1–0.3)	0.007
D4	0.4 (0.2–0.8)	0.25 (0.2–0.3)	0.009
D5	0.4 (0.2–0.8)	0.2 (0.1–0.3)	0.001
D6	0.3 (0.1–0.6)	0.2 (0.1–0.3)	0.022
D7	0.4 (0.2–1.0)	0.3 (0.2–0.37)	0.013
D8	0.4 (0.2–0.8)	0.2 (0.1–0.3)	0.001
D9	0.5 (0.3–1.1)	0.2 (0.2–0.3)	<0.001
D10	0.6 (0.3–0.9)	0.25 (0.1–0.3)	<0.001
AD1	0.3 (0.1–0.5)	0.2 (0.1–0.3)	0.138
AD2	0.3 (0.1–0.6)	0.2 (0.1–0.3)	0.005
RADIAL	0.7 (0.2–0.8)	0.2 (0.1–0.3)	<0.001
ULNAR	0.5 (0.2–0.9)	0.2 (0.1–0.3	<0.001
MEDIAN	0.4 (0.1–0.7)	0.2 (0.1–0.3)	<0.001
**FEET**			
PL1	0.5 (0.3–1.3)	0.2 (0.1–0.3)	<0.001
PL2	0.7 (0.4–1.3)	0.2 (0.02–0.3)	<0.001
PL3	0.8 (0.3–1.3)	0.2 (0.1–0.3)	<0.001
PL4	0.6 (0.3–1.0)	0.25 (0.1–0.4)	0.001
PL5	0.7 (0.4–1.3)	0.2 (0.1–0.3)	<0.001
PL6	0.5 (0.2–0.8)	0.2 (0.2–0.3)	0.003
PL7	0.5 (0.3–0.8)	0.2 (0.1–0.3)	<0.001
PL8	0.4 (0.2–0.8)	0.2 (0.1–0.3)	0.001
PL9	0.5 (0.2–0.8)	0.2 (0.1–0.3)	<0.001
PL10	0.4 (0.2–0.7)	0.2 (0.1–0.3)	<0.001
PF	0.4 (0.2–0.8)	0.2 (0.1–0.3)	<0.001
D1	0.5 (0.2–1.3)	0.15 (0.1–0.3)	<0.001
D2	0.4 (0.2–1.0)	0.2 (0.1–0.3)	<0.001
D3	0.5 (0.2–0.8)	0.2 (0.2–0.3)	<0.001
D4	0.4 (0.2–0.9)	0.2 (0.1–0.3)	0.005
D5	0.7 (0.3–1.3)	0.2 (0.1–0.3)	<0.001
AD1	0.4 (0.1–0.9)	0.15 (0.1–0.3)	0.009
AD2	0.4 (0.1–0.7)	0.15 (0.1–0.3	0.005
FIBULAR	0.6 (0.3–1.1)	0.2 (0.1–0.3)	<0.001
TIBIAL	0.5 (0.2–1.0)	0.2 (0.1–0.3)	<0.001

ROI: Region of interest, *p-value comparison between ΔTs of patients and controls

Mann- Whitney test. * p was considered significant when p < 0.05.

When the groups of patients were compared to each other, the average temperature in the hands of the PWP group ranged from 31.38°C to 33.63°C and in the PNP group from 31.71°C to 33.6°C. In the feet, it varied from 29.62°C to 32.01°C in the PWP group and 29.35°C to 32.11°C in the PNP group. In the PWP group, there was a significant difference in the mean temperature on the right side with the left in two ROIs (P2 and AD2) in the hands and two ROIs (PL2 and PL7) in the feet (p < 0.05). In the PNP group, there was no significant difference between the average temperatures (p > 0.05) (S2 Table). There was also no significant difference of ΔT in the ROIs and neural areas of the patients’ hands and feet. (Table 3). In the hands of the PWP group, the median of ΔT varied from 0.3°C to 0.7°C in the hands and 0.3 to 0.8°C in the feet. In the PNP group, there was a median variation from 0.2°C to 0.6°C in hands and 0.3°C to 0.75°C in the feet. Although the asymmetry is slightly higher in the PWP group, the values were not significant (Table 3).

**Table 3:** Comparation of ΔT in the ROIs and neural areas of hands and feet in the PWP and PNP groups.

	PWP (n = 29)	PNP (n = 26)	
ROI	ΔT	ΔT	
	median (Q1-Q3)	median (Q1-Q3)	*p value*
**HANDS**			
P1	0.7 (0.35–1.15)	0.5 (0.3–1.15)	0.191
P2	0.5 (0.2–0.8)	0.4 (0.2–0.7)	0.551
P3	0.6 (0.35–1.3)	0.3 (0.2–0.7)	0.677
P4	0.5 (0.2–1.0)	0.3 (0.2–0.5)	0.295
P5	0.6 (0.25–1.0)	0.5 (0.3--0.75)	0.878
P6	0.6 (0.3–1.2)	0.2 (0.2–0.8)	0.060
P7	0.6 (0.2–1.05)	0.45 (0.2–1.15)	0.754
P8	0.5 (0.25–0.85)	0.5 (0.2–1.07)	0.388
P9	0.6 (0.4–1.15)	0.5 (0.2–1.15)	0.869
P10	0.4 (0.3–1.05)	0.35 (0.2–1.1)	0.883
AP1	0.3 (0.15–0.8)	0.2 (0.1–0.75)	0.116
AP2	0.3 (0.2–0.65)	0.3 (0.1–0.75)	0.638
D1	0.6 (0.25–1.2)	0.35 (0.1–0.85)	0.162
D2	0.5 (0.3–1.3)	0,25 (0.1–0.5)	0.061
D3	0.4 (0.15–1.2)	0.4 (0.1–0.9)	0.708
D4	0.3 (0.15–0.7)	0.45 (0.4–0.9)	0.876
D5	0.6 (0.2–0.9)	0.45 (0.2–0.7)	0.398
D6	0.3 (0.1–0.6)	0.4 (0.1–0.6)	0.303
D7	0.5 (0.2–1.05)	0.4 (0.2–0.9)	0.334
D8	0.4 (0.2–1.0)	0.35 (0.2–0.7)	0.324
D9	0.5 (0.25–1.2)	0.55 (0.3–1.1)	0.871
D10	0.6 (0.25–1.0)	0.6 (0.5–0.9)	0.876
AD1	0.2 (0.1–0.5)	0.3 (0.2–0.6)	0.644
AD2	0.6 (0.25–1.0)	0.3 (0.2–0.7)	1
RADIAL	0.4 (0.2–0.65)	0.35 (0.2–0.6)	0.760
ULNAR	0.2 (0.3–0.7)	0.45 (0.2–0.8)	0.684
MEDIAN	0.5 (0.2–1.0)	0.4 (0.3–1.0)	0.618
**FEET**			
PL1	0.5 (0.25–1.4)	0.6 (0.4–1.15)	0.848
PL2	0.8 (0.4–1.4)	0.4 (0.3–1.1)	0.064
PL3	0.8 (0.4–1.2)	0.7 (0.30–1.4)	0.896
PL4	0.7 (0.3–1.1)	0.55 (0.20–1.0)	0.486
PL5	0.7 (0.45–1.25)	0.6 (0.3--1.3)	0.640
PL6	0.6 (0.2–0.95)	0.45 (0.2–0.7)	0.613
PL7	0.6 (0.3–0.85)	0.4 (0.3–0.9)	0.908
PL8	0.5 (0.25–0.8)	0.4 (0.2–0.8)	0.753
PL9	0.4 (0.25–1.05)	0.5 (0.2–0.7)	0.976
PL10	0.5 (0.2–0.75)	0.3 (0.2–0.6)	0.369
PF	0.6 (0.35–1.15)	0.4 (0.2–0.7)	0.165
D1	0.7 (0.2–1.55)	0.4 (0.2–0.9)	0.207
D2	0.4 (0.2–1.05)	0.4 (0.3–1.0)	0.495
D3	0.5 (0.2–1.1)	0.45 (0.3–0.8)	0.878
D4	0.3 (0.1–0.9)	0.5 (0.3–1.1)	0.098
D5	0.7 (0.3–1.35)	0.75 (0.3–1.3)	0.713
AD1	0.6 (0.1–1.1)	0.4 (0.1–0.6)	0.477
AD2	0.5 (0.2–0.6)	0.45 (0.1–0.8)	0.485
FIBULAR	0.5 (0.15–1.0)	0.4 (0.3–0.7)	0.903
TIBIAL	0.6 (0.3–1.0)	0.45 (0.2–0.8)	0.516

ROI: Region of interest, PWP: patients with pain, PNP: patients no pain.

ΔT: right/left temperature difference; * p was considered significant when p < 0.05 (Mann-Whitney test).

## Discussion

NP in leprosy has been underdiagnosed and poorly treated. Therefore, new tools are needed to help in the correct approach to the diagnosis of pain, because the presence of pain greatly impacts the quality of life of patients [8]. Patients with leprosy relapse and treatment failure have a greater chance of neural complications such as NP, because they are a group of patients chronically affected by the disease [29]. The population of this study was composed mostly of individuals in the economically active age group, with a mean age of 46.75 (±11.4) years in the PWP group and 48 (±13.4) years in the PNP group, showing the high social impact of leprosy [30,31]. There was a predominance of males among the patients. According to a systematic review study, men are at a higher risk of acquiring leprosy, while women are probably more concerned about their health and are diagnosed early, and/or there are different levels of leprosy exposure risk factors in men and women [32]. According to the WHO operational classification, 81.8% (45/55) were multibacillary patients, and the clinical forms with the highest incidence were lepromatous and borderline-tuberculoid, as the region is highly endemic, and this result corroborates the epidemiological results [33].

In the present study, 92.7% (51/55) of patients had previous reactional episodes, and the PWP group had a higher number of type 1 reactions than the PNP group (p = 0.02). Multibacillary patients have a higher chance of developing leprosy reactions regardless of the type [34]. Type 1 reaction is the main cause of neural involvement in leprosy [35] and is accompanied by neuritis associated with sensory and motor changes. Leprosy reactions are significant risk factors for the triggering of NP [36].

It has been described that sensory and autonomic fibers (small-fiber neuropathy) are the first to be affected in leprosy [37], therefore, when motor changes occur, it is a sign that the disease is in an advanced phase with involvement of several nerve trunks [2], leading to the classic pattern in the electroneurophysiological examination of multiple sensori-motor asymmetric mononeuritis [38]. In this study, most patients were already in an advanced stage of the disease, especially those in the PWP group, with significant motor loss (p = 0.001) and a predominance of multiple asymmetric mononeuritis (p = 0.045). The highest degree of disability was related to pain and the general poor perception of health [5]; although no significant difference was shown between the groups, the prevalence of disability grade II was high (37.9%) in the PWP group. These findings confirm that NP is a late complication of leprosy [39].

In this study, the prevalence of NP was 52.7% (29/55). The high prevalence of NP in patients with relapse and treatment failure could be caused by chronic inflammation around the nerve, the existence of persistent *M leprae* antigens [37], or resistant drugs [29]. In the Reference Center, all cases of relapse and therapeutic failure were investigated for drug resistance, and no drug resistance was found. The early diagnosis of neuropathy in leprosy relapse has been defiant due to the long incubation period of the bacillus and the presence of sequelae that hinder the differential diagnosis [28].

Regarding pain localization, this study showed a higher incidence in the tibial and fibular nerves, a fact that differed from other studies that reported a higher incidence in the ulnar nerves [5,40]. Tibial nerve neuropathy may be underdiagnosed, as tibial neuritis may develop silently [41]. Chronic pain is defined as persistent or recurrent for more than or equal to 3 months [42], and it is known that the neuropathic component in chronic pain impacts more on the quality of life [10] than pain without the neuropathic component [43]. All individuals with NP presented chronic pain intermittently or continuously and 93% reported moderate to severe pain. These findings show the chronic suffering to which patients are submitted and the lack of diagnosis of NP [42]. Given that the sampling of this study was randomly obtained, it was not possible to compare individuals with neuropathic and nociceptive pain, which is a limitation of the study.

The IRT has been used as an auxiliary tool in the triage and monitoring of neuropathic painful syndromes, but its use should be judicious because it reflects the emission of heat determined by physiological and pathological neural processes or not [44]. The skin temperature difference in symmetrical areas of the body may indicate a pathologic process [45]. In neural lesions, hypothermic or hyperthermic variations that are reflected in thermal imaging may occur and can be valuable in the diagnosis and proper treatment of pain [46].

In this study, the average temperature of the ROIs showed almost no significant difference between the control group and leprosy patients (PWP and PNP), which due to the subtle variation in the temperature distribution [46] or because of leprosy, neuropathy occurs asymmetrically and to varying degrees between patients, and it is not possible to determine a single temperature pattern. The temperatures of the hands and feet varied throughout the day between individuals and in the individual himself, being less stable than the other parts of the body, but the ΔT remained constant in the same individual [47].

In this study, the method used was the static method, where the temperature measurement was performed in a single moment and the main way of evaluating the temperatures in this method was by ΔT, which evaluates the temperature difference on one side of the body compared to its contralateral side [48]. There is a thermal symmetry between the areas of the human body, where the temperature difference of < 0.3°C between the areas of the body is considered normal [47,49]. Asymmetry (ΔT) was significant in all ROIs (hands and feet) evaluated when all leprosy patients were compared with the controls, except for the palm and dorsal points, indicating once again the asymmetric characteristic of leprosy and the greater distal neural involvement of the hands.

There was no statistical difference in the ΔT between the PWP and PNP groups in the ROIs and neural areas evaluated, probably because most patients were already presenting an advanced neural disease. The static thermography was able to diagnose autonomic dysfunction in leprosy, which is not always accompanied by NP; therefore, thermal images should always be analyzed based on clinical evaluation. It should be considered that thermography captures acute or chronic sympathetic nervous system dysfunction, and as chronic neurological disease progresses, sympathetic thermal dysfunction becomes bilateral [49]. Thermography may indicate a change in abnormal physiology, but it is nonspecific, and its thermal patterns must be properly interpreted in order not to lead to diagnostic error [48].

IRT seems to be a promising method to approach pain, as it is safe and non-invasive. By tracking changes in the microcirculation of the hands and feet of patients with leprosy, sensorimotor neural assessments can be correlated with the dysautonomia demonstrated by the skin’s thermal pattern.

The creation of a database with individual thermograms can assist in monitoring the progression of neuropathies through temperature changes. These repeated accompanying measures during treatment will clarify the link between asymmetric temperature distributions and pathophysiological changes on the skin surface and the extent of the neural injury.

Given its limitations, IRT cannot be considered as a substitute for clinical examination and motor-sensory evaluation, but it complements this peripheral neural evaluation and can assist in therapeutic approaches to pain, impacting measures to prevent leprosy deficiencies.

PN in leprosy needs to be better understood to be treated properly, as it directly impacts the individual’s quality of life. Further IRT studies are needed, evaluating the patient early before the installation of permanent neural damage and using dynamic methods associated with thermal stress tests that can increase sensitivity for the detection of NP dysautonomia in leprosy, thereby allowing for the better understanding and applicability of thermal tests.

## Supporting information

S1 Table Comparison of mean temperature in ROIs and neural areas of the hands between the group of patients and healthy controls. (DOCX)

S2 Table Comparison of average temperature in ROIs and neural areas of hands and feet in the group of PWP and PNP. (DOCX)
