# Supplementary material for: Assessment of neuropathic pain in leprosy patients with relapse or treatment failure by infrared thermography: A cross-sectional study
Source: PLoS Negl Trop Dis. 2021 Sep 23;15(9):e0009794. doi: 10.1371/journal.pntd.0009794 (PMC8491942; doi:10.1371/journal.pntd.0009794)
Supplement: S1 Table — (DOCX) [file pntd.0009794.s001.docx]

**S1Table****: Comparison of average temperature in ROIs and neural areas of hands and feet in the group of leprosy patients (PWP and PNP) and healthy control.**

|  | **LEPROSY PATIENTS**  **(n=55)** | | | **HEALTHY CONTROLS**  **(n=20)** | |  |
| --- | --- | --- | --- | --- | --- | --- |
| **ROI** | **Right** | **Left** |  | **Right** | **Left** |  |
|  | mean (SD) (ºC) | mean (SD) (ºC) | *p value* | mean (SD) (ºC) | mean (SD) (ºC) | *p value* |
| **HANDS** |  |  |  |  |  |  |
| P1 | 32.82 ± 2.85 | 32.55 ± 3.12 | 0.256 | 33.13 ± 1.49 | 33.17 ± 1.40 | 0.353 |
| P2 | 33.33 ± 2.24 | 33.11 ± 2.42 | 0.280 | 33.21 ± 1.41 | 33.19 ± 1.42 | 0.331 |
| P3 | 32.65 ± 3.00 | 32.28 ± 3.27 | 0.078 | 33.02 ± 1.75 | 33.03 ± 1.73 | 0.337 |
| P4 | 32.88 ± 2.50 | 32.70 ± 2.64 | 0.023* | 33.04 ± 1.70 | 33.09 ± 1.70 | 0.290 |
| P5 | 32.38 ± 3.00 | 32.03 ± 3.10 | 0.002* | 32.86 ± 1.58 | 32.95 ± 1.60 | 0.035* |
| P6 | 33.03 ± 2.44 | 32.90 ± 2.48 | 0.591 | 33.09 ± 1.62 | 32.98 ± 1.67 | 0.100 |
| P7 | 32.32 ± 2.99 | 31.92 ± 3.32 | 0.097 | 32.75 ± 1.60 | 32.80 ± 1.66 | 0.834 |
| P8 | 32.88 ± 2.58 | 32.75 ± 2.46 | 0.118 | 32.97 ± 1.60 | 33.04 ± 1.64 | 0.267 |
| P9 | 31.91 ± 2.87 | 31.69 ± 3.32 | 0.831 | 32.61 ± 1.84 | 32.59 ±1.87 | 0.587 |
| P10 | 32.67 ± 2.43 | 32.41 ± 2.72 | 0.003* | 32.81 ± 1.76 | 32.73 ± 1.79 | 0.160 |
| AP1 | 33.74 ± 1.74 | 33.67 ± 1.86 | 0.306 | 33.66 ± 1.43 | 33.71 ± 1.33 | 0.428 |
| AP2 | 33.66 ± 1.74 | 33.57 ± 1.80 | 0.000* | 33.62 ± 1.45 | 33.68 ± 1.36 | 0.330 |
| D1 | 32.84 ± 2.94 | 32.51 ± 3.13 | 0.206 | 33.01 ± 1.81 | 32.91 ± 1.79 | 1.0 |
| D2 | 33.10 ± 2.39 | 33.00 ± 2.41 | 0.742 | 32.90 ± 1.70 | 32.82 ± 1.71 | 0.145 |
| D3 | 32.55 ± 2.91 | 32.28 ± 2.96 | 0.194 | 32.80 ± 1.89 | 32.69 ± 1.85 | 0.163 |
| D4 | 32.40 ± 2.54 | 32.35 ± 2.37 | 0.496 | 32.62 ± 1.80 | 32.58 ± 1.85 | 0.481 |
| D5 | 32.37 ± 2.91 | 32.25 ± 2.87 | 0.795 | 32.79 ± 1.82 | 32.83. ± 1.85 | 1 |
| D6 | 32.45 ± 2.48 | 32.46 ± 2.35 | 0.732 | 32.64 ± 1.73 | 32.59 ± 1.75 | 0.349 |
| D7 | 32.34 ± 2.92 | 32.13 ± 2.97 | 0.428 | 32.70 ± 2.00 | 32.65 ± 1.94 | 0.330 |
| D8 | 32.37 ± 2.50 | 32..33 ± 2.39 | 0.725 | 32.64 ± 1.86 | 32.60 ± 1.79 | 0.716 |
| D9 | 31.94 ± 2.88 | 31,90 ± 2.94 | 1 | 32.44 ± 2.10 | 32.37 ± 2.01 | 0.312 |
| D10 | 32.01 ± 2.52 | 32.15 ± 2.45 | 0.209 | 32.36 ± 1.88 | 32.34 ± 1.88 | 0.733 |
| AD1 | 33.06 ± 1.78 | 33.07 ± 1.60 | 0.306 | 32.89 ± 1.58 | 32.86 ± 1.52 | 0.609 |
| AD2 | 32.91 ± 1.87 | 32.90 ± 1.78 | 0.398 | 32.89 ± 1.51 | 32.79 ± 1.51 | 0.577 |
| RADIAL | 32.51 ± 0.69 | 32.43 ± 0.69 | 0.424 | 32.78 ± 0.12 | 32.75 ± 0.49 | 0.488 |
| ULNAR | 32.85 ± 0.48 | 32.41 ± 0.34 | 0.338 | 33.06 ± 0.14 | 33.1 ± 0.25 | 0.491 |
| MEDIAN | 32.75 ± 0.37 | 32.72 ± 0.36 | 0.224 | 32.76 ± 0.15 | 32.71 ± 0.14 | 0.819 |
| PL1 | 30.12 ± 2.60 | 30.04 ± 2.69 | 0.370 | 30.92 ± 2.24 | 30.93 ± 2.29 | 0.666 |
| PL2 | 29.50 ± 2.71 | 29.72 ± 2.83 | 0.100 | 30.50 ± 2.45 | 30.53 ± 2.48 | 0.330 |
| PL3 | 29.60 ± 2.66 | 29.69 ± 2.80 | 0.219 | 30.60 ± 2.38 | 30.56 ± 2.49 | 0.660 |
| PL4 | 29.71 ± 2.60 | 29.83 ± 2.71 | 0.336 | 30.38 ± 2.26 | 30.41 ± 2.38 | 0.163 |
| PL5 | 29.84 ± 2.54 | 29.86 ± 2.47 | 0.430 | 30.43 ± 2.11 | 30.38 ± 2.21 | 1.0 |
| PL6 | 30.77 ± 2.16 | 30.68 ± 2.27 | 0.147 | 31.11 ± 1.66 | 31.11±1.69 | 0.876 |
| PL7 | 30.67 ± 2.14 | 30.45 ± 2.15 | 0.038* | 30.66 ± 1.66 | 30.59 ± 2.00 | 0.330 |
| PL8 | 31.61 ± 1.74 | 31.48 ± 1.74 | 0.031* | 31.69 ± 0.97 | 31.69 ± 1.07 | 1.0 |
| PL9 | 30.65 ± 1.96 | 30.62 ± 1.94 | 0.874 | 30.75 ± 1.61 | 30.72 ± 1.59 | 0.579 |
| PL10 | 30.49 ± 2.26 | 30.51 ± 2.21 | 0.416 | 30.79 ± 1.45 | 30.78 ± 1.51 | 0.592 |
| F | 31.54 ± 2.13 | 31.55 ± 2.17 | 0.632 | 31.86 ± 1.95 | 31.85 ± 1.94 | 0.293 |
| D1 | 31.08 ± 2.50 | 31.00 ± 2.48 | 0.805 | 31.34 ± 1.93 | 31.28 ± 1.96 | 0.423 |
| D2 | 30.70 ± 2.39 | 30.81 ± 2.46 | 0.000* | 31.19 ± 2.05 | 31.13 ± 1.98 | 0.330 |
| D3 | 30.69 ± 2.42 | 30.89 ± 2.50 | 0.026* | 31.31 ± 1.96 | 31.28 ± 1.89 | 0.085 |
| D4 | 30.69 ± 2.42 | 30.84 ± 2.46 | 0.228 | 31.27 ± 2.00 | 31.25 ± 1.95 | 0.666 |
| D5 | 30.59 ± 2.44 | 30.68 ± 2.31 | 0.370 | 31.09 ± 1.90 | 31.03 ± 1.91 | 0.473 |
| AD1 | 32.03 ± 1.72 | 31.82 ± 1.85 | 0.070 | 32.11 ± 1.37 | 32.09 ± 1.34 | 0.310 |
| AD2 | 32.06 ± 1.72 | 32.02 ± 1.79 | 1.0 | 32.12 ± 1.21 | 32.15 ± 1.24 | 0.577 |
| FIBULAR | 31.15 ± 0.59 | 31.22 ± 0.51 | 0.277 | 31.53 ± 0.37 | 31.5 ± 0.44 | 0.331 |
| TIBIAL | 30.29 ± 0.68 | 30.28 ± 0.56 | 0.251 | 30.78 ± 0.38 | 30.77 ± 0.39 | 0.330 |

ROI: Region of interest, PWP: patients with pain, PNP: patients no pain, *p-value* comparison between right and left side.**p-value* was considered significant when p<0.05 (Mann-Whitney test).
