# Supplementary material for: Assessment of neuropathic pain in leprosy patients with relapse or treatment failure by infrared thermography: A cross-sectional study
Source: PLoS Negl Trop Dis. 2021 Sep 23;15(9):e0009794. doi: 10.1371/journal.pntd.0009794 (PMC8491942; doi:10.1371/journal.pntd.0009794)
Supplement: S2 Table — (DOCX) [file pntd.0009794.s002.docx]

| **ROI** | **PWP**  **(n=29)** | |  | **PNP**  **(n=26)** | |  |
| --- | --- | --- | --- | --- | --- | --- |
|  | **Right** | **Left** |  | **Rigth** | **Left** |  |
|  | mean (SD) (ºC) | mean (SD) (ºC) | **p value* | mean (SD) (ºC) | mean (SD) (ºC) | **p value* |
| **HANDS** |  |  |  |  |  |  |
| P1 | 32.48 ± 3.11 | 32.33 ± 3.32 | 0.256 | 32.89 ± 2.91 | 32.61 ± 2.97 | 0.611 |
| P2 | 33.12 ± 2.41 | 32.85 ± 2.68 | 0.031* | 33.30 ± 2.41 | 33.25 ± 2.11 | 0.570 |
| P3 | 32.27 ± 3.16 | 32.03 ± 3.38 | 0.174 | 32.81 ± 2.90 | 32.31 ± 3.19 | 0.179 |
| P4 | 32.65 ± 2.57 | 32.41 ± 2.79 | 0.110 | 32.88 ± 2.58 | 32.83 ± 2.48 | 0.368 |
| P5 | 32.11 ± 3.05 | 31.82 ± 3.22 | 0.053 | 32.38 ± 3.12 | 32.03 ± 3.03 | 0.903 |
| P6 | 32.85 ± 2.45 | 32.68 ± 2.53 | 0.056 | 33.0 ± 2.60 | 32.95 ± 2.45 | 0.866 |
| P7 | 32.02 ± 3.06 | 31.59 ± 3.46 | 0.201 | 32.28 ± 3.26 | 32.04 ± 3.23 | 0.547 |
| P8 | 32.71 ± 2.47 | 32.45 ± 2.57 | 0.892 | 32.84 ± 2.75 | 32.88 ± 2.32 | 0.107 |
| P9 | 31.50 ± 2.81 | 31.38 ± 3.35 | 0.518 | 31.90 ± 3.32 | 31.71 ± 3.31 | 0.279 |
| P10 | 32.28 ± 2.40 | 32.20 ± 2.54 | 0.720 | 32.72 ± 2.80 | 32.34 ± 3.06 | 0.645 |
| AP1 | 33.63 ± 1.72 | 33.49 ± 1.95 | 0.168 | 33.61 ± 1.99 | 33.76 ± 1.67 | 0.409 |
| AP2 | 33.53 ± 1.68 | 33.38 ± 1.74 | 0.199 | 33.48 ± 2.11 | 33.60 ± 1.81 | 0.601 |
| D1 | 32.52 ± 3.23 | 31.93 ± 3.54 | 0.168 | 32.88 ± 3.01 | 32.88 ± 2.64 | 0.947 |
| D2 | 32.88 ± 2.58 | 32.69 ± 2.64 | 0.291 | 33.08 ± 2.46 | 33.09 ± 2.17 | 0.815 |
| D3 | 32.13 ± 3.25 | 31.87 ± 3.0 | 0.733 | 32.65 ± 2.78 | 32.48 ± 2.98 | 0.725 |
| D4 | 32.01 ± 2.77 | 31.95 ± 2.42 | 0.251 | 32.53 ± 2.50 | 32.58 ± 2.29 | 0.662 |
| D5 | 32.04 ± 3.06 | 31.85 ± 2.93 | 0.973 | 32.36 ± 3.07 | 32.47 ± 2.83 | 0.923 |
| D6 | 32.08 ± 2.73 | 32.0 8± 2.42 | 0.129 | 32.51 ± 2.47 | 32.66 ± 2.23 | 0.520 |
| D7 | 32.0 ± 3.05 | 31.65 ± 3.03 | 0.180 | 32.33 ± 3.18 | 32.41 ± 2.92 | 0.648 |
| D8 | 32.01 ± 2.66 | 31.93 ± 2.41 | 0.694 | 32.42 ± 2.63 | 32.51 ± 2.34 | 0.542 |
| D9 | 31.56 ± 2.84 | 31.46 ± 2.92 | 0.221 | 31.95 ± 3.23 | 32.09 ± 2.94 | 0.662 |
| D10 | 31.56 ± 2.57 | 31.81 ± 2.45 | 0.510 | 32.16 ± 2.71 | 32.25 ± 2.43 | 0.962 |
| AD1 | 32.75 ± 1.78 | 32.86 ± 1.53 | 0.442 | 33.12 ± 1.99 | 33.15 ± 1.62 | 0.776 |
| AD2 | 32.58 ± 1.85 | 32.66 ± 1.68 | 0.013* | 32.91 ± 2.20 | 32.98 ± 1.82 | 0.841 |
| RADIAL | 32.63 ± 0.41 | 32.60 ± 0.42 | 0.425 | 32.81 ± 0.32 | 32.64 ± 0.29 | 0.646 |
| ULNAR | 32.39 ± 0.75 | 32.38 ± 0.71 | 0.286 | 32.52 ± 0.68 | 32.49 ± 0.68 | 0.802 |
| MEDIAN | 32.78 ± 0.45 | 32.42 ± 0.50 | 0.180 | 32.87 ± 0.38 | 32.71 ± 0.49 | 0.810 |
| **FEET** |  |  |  |  |  |  |
| PL1 | 30.04 ± 2.38 | 30.08 ± 2.64 | 0914 | 29.93 ± 2.84 | 29.73 ± 2.80 | 0.393 |
| PL2 | 30.40 ± 2.48 | 29.78 ± 2.84 | 0.041* | 29.29 ± 2.89 | 29.72 ± 3.23 | 0.249 |
| PL3 | 29.66 ± 2.62 | 29.89 ± 2.82 | 0.397 | 29.37 ± 2.76 | 29.35 ± 2.89 | 0.672 |
| PL4 | 29.62 ± 2.54 | 29.87 ± 2.66 | 0.117 | 29.53 ± 2.71 | 29.66 ± 2.89 | 0.397 |
| PL5 | 29.93 ± 2.55 | 29.89 ± 2.46 | 0.660 | 29.53 ± 2.58 | 29.68 ± 2.59 | 0.107 |
| PL6 | 30.69 ± 2.09 | 30.69 ± 2.27 | 0.876 | 30.60 ± 2.28 | 30.43 ± 2.33 | 0.027 |
| PL7 | 30.68 ± 2.19 | 30.45 ± 2.19 | 0.038* | 30.44 ± 2.10 | 30.25 ± 2.16 | 0.198 |
| PL8 | 31.50 ± 1.72 | 31.34 ± 1.69 | 0.192 | 31.55 ± 1.73 | 31.46 ± 1.78 | 0.238 |
| PL9 | 30.64 ± 2.11 | 30.58 ± 2.03 | 0.579 | 30.46 ± 1.81 | 30.51 ± 1.87 | 0.819 |
| PL10 | 30.53 ± 2.30 | 30.51 ± 2.32 | 0.293 | 30.25 ± 2.33 | 30.39 ± 2.20 | 0.195 |
| F | 31.62 ± 2.14 | 31.39 ± 2.41 | 0.423 | 31.35 ± 2.19 | 31.36 ± 2.29 | 0.622 |
| D1 | 31.04 ± 2.41 | 30.89 ± 2.40 | 0.203 | 30.81 ± 2.65 | 30.82 ± 2.67 | 0.741 |
| D2 | 30.62 ± 2.31 | 30.85 ± 2.42 | 0.085 | 30.56 ± 2.53 | 30.54 ± 2.58 | 0.573 |
| D3 | 30.64 ± 2.43 | 30.97 ± 2.51 | 0.455 | 30.51 ± 2.50 | 30.60 ± 2.61 | 0.585 |
| D4 | 30.64 ± 2.39 | 30.82 ± 2.38 | 0.473 | 30.55 ± 2.56 | 30.71 ± 2.67 | 0.381 |
| D5 | 30.51 ± 2.49 | 30.70 ± 2.23 | 0.310 | 30.46 ± 2.44 | 30.51 ± 2.50 | 0.958 |
| AD1 | 31.93 ± 1.68 | 31.90 ± 1.79 | 0.070 | 31.98 ± 1.82 | 31.71 ± 1.96 | 0.292 |
| AD2 | 31.88 ± 1.67 | 32.01 ± 1.71 | 0.398 | 32.11 ± 1.73 | 31.91 ± 1.91 | 0.707 |
| FIBULAR | 31.27 ± 0.56 | 31.28 ± 0.47 | 0.397 | 31.04 ± 0.68 | 31.02 ± 0.55 | 0.695 |
| TIBIAL | 30.55 ± 0.59 | 30.43 ± 0.51 | 0.677 | 30.09 ± 0.70 | 30.16 ± 0.63 | 0.835 |

**S2 Table: Comparison of average temperature in ROIs and neural areas of hands and feet in the group of PWP and PNP.**

ROI: Region of interest, PWP: patients with pain, PNP: patients no pain, * p-value comparison between right and left side. **p-value comparison between average temperature of patients with pain and patients no pain. p- value was considered significant when p < 0.05 (Mann-Whitney test).
